# Influence of Lifestyle Factors on Ocular Surface Parameters in Relation to Age

**DOI:** 10.3390/life14111460

**Published:** 2024-11-11

**Authors:** Jacobo Garcia-Queiruga, Hugo Pena-Verdeal, Belen Sabucedo-Villamarin, Veronica Noya-Padin, Maria J. Giraldez, Eva Yebra-Pimentel

**Affiliations:** 1 GI-2092 Optometry, Departamento de Física Aplicada, Facultad de Óptica y Optometría, Universidade de Santiago de Compostela, Campus Vida s/n, 15701 Santiago de Compostela, Spain; belen.sabucedo@rai.usc.es (B.S.-V.); veronicanoya.padin@usc.es (V.N.-P.); mjesus.giraldez@usc.es (M.J.G.); eva.yebra-pimentel@usc.es (E.Y.-P.); 2 AC-24 Optometry, Instituto de Investigación Sanitaria de Santiago de Compostela (IDIS), Travesía da Choupana, 15701 Santiago de Compostela, Spain

**Keywords:** lifestyle, tear film osmolarity, FBUT, VDT, contact lens, age, eyedrop

## Abstract

Purpose: To evaluate how Video Display Terminal (VDT) use, Contact Lens (CL) wear, and eyedrop use affect ocular surface parameters in participants with ocular discomfort and how these factors may vary across different age groups. Methods: The current cross-sectional study initially involved a total of 252 participants who completed a self-administered survey to collect information about ocular discomfort and lifestyle factors. This online survey was composed of an Ocular Surface Disease Index (OSDI) questionnaire and three extra questions regarding lifestyle factors (VDT use, CL wear, and eyedrop use). Only 185 symptomatic participants, those with OSDI values > 12, were scheduled to undergo a comprehensive ocular examination that included tear film osmolarity, Fluorescein Break-Up Time (FBUT), Maximum Blink Interval (MBI), and corneal staining. Results: Differences in ocular parameters and lifestyle factors across age groups (<40 years, 40–60 years, >60 years) were analyzed, along with their correlations and regression. Significant age group differences were found in OSDI, osmolarity, FBUT, and MBI (One-way ANOVA, all *p* ≤ 0.029). Correlations were observed between CL wear and osmolarity and MBI (Pearson’s correlation, both *p* ≤ 0.049). Multiple regression confirmed age associations with OSDI, osmolarity, FBUT, and MBI (Multiple linear regression, all *p* ≤ 0.040) and found links between VDT use and osmolarity and MBI (Multiple linear regression, both *p* ≤ 0.038) and between eyedrop use and OSDI (Multiple linear regression, *p* = 0.040). Conclusion: Aging is a primary factor affecting ocular homeostasis, with older adults showing lower FBUT and MBI values and higher osmolarity. Prolonged use of VDTs exacerbates this effect, further contributing to ocular discomfort and destabilized tear film. No associations between CL wear and any of the ocular parameters were found. Eyedrop use shows varied effects on ocular comfort across age groups, emphasizing the need for age-specific ocular care. Overall, these findings confirm that aging and extended VDT use play a significant role in ocular surface discomfort.

## 1. Introduction

The tear film is a thin layer covering the entire epithelium of the ocular surface, including both the cornea and conjunctiva [1]. The tear film and ocular surface together compose the Lacrimal Function Unit (LFU), a complex system responsible for the protection, moisture, and nutrition of ocular tissues [1,2]. An alteration in any of the tissues responsible for tear film production (lacrimal glands, meibomian glands, or globe cells located in the conjunctival mucosa) or in the spreading of the tear film over the ocular surface (reduced blink rate, incomplete blinking, or eyelids malposition) triggers an inflammatory cascade with consequent compensatory events such as increased tear production o reflex blinking [3,4]. However, when there is continuous exposure to these triggers or excessive stress on the LFU, it results in a vicious cycle led by hyperosmolarity due to the high presence of inflammatory mediators [3,4]. A patient in this situation usually manifests symptoms of ocular discomfort such as blurred vision, burning, itching, or gritty feeling in their eyes [5,6,7].

In 2023, the Tear Film and Ocular Surface Society published a report on the lifestyle factors that may impact ocular health [8]. This workshop collected available data about how lifestyle factors could affect different ocular structures [9]. There are factors that the sufferer could manage, such as excessive use of Video Display Terminals (VDT) (computer, smartphone, and tablet) or wearing Contact Lenses (CL) for periods longer than recommended, among others [5,6,7,9,10]. Prolonged exposure to VDT has been documented as reducing tear film stability, reducing blinking rate, and increasing the number of incomplete blinks while using them [6,11]. CL wearers had lower tear film break-up time values and higher ocular discomfort values on various symptomatology questionnaires compared to non-CL wearers [10,12]. On the other hand, there are intrinsic factors that a patient cannot manage, such as aging or suffering from different systemic diseases that affect ocular health (i.e., thyroid disease or rheumatoid arthritis), among others [9,13]. Overall, when ocular discomfort arises, whether due to prolonged VDT use, contact lens wear, or ocular diseases such as dry eye, its management typically involves prescribing eyedrops to restore ocular homeostasis; in this situation, using eyedrops can be a lifestyle factor with both extrinsic and intrinsic aspects, and it may also serve as a protective measure.

Age is an important factor to monitor in cross-sectional studies, as numerous age-related changes throughout the body have been documented, such as muscle loss, hearing loss, visual loss, and decreased immune function [14,15]. Regarding the eye, visual loss is often due to presbyopia, which is common among people older than 40 years [16,17]. The loss of near vision due to presbyopia affects daily activities, reducing not only those activities that involve near vision but also those that require spectacles or CL for clear vision at both distances, such as practicing ball or racket sports [16,17,18].

Considering this context, the aim of the present study was to assess how VDT use, CL wear, and eyedrop use influence ocular surface parameters in a sample of participants with ocular discomfort segregated by age. Additionally, the study aimed to explore how these lifestyle factors vary across different age groups.

## 2. Materials and Methods

### 2.1. Sample

**Figure 1:**
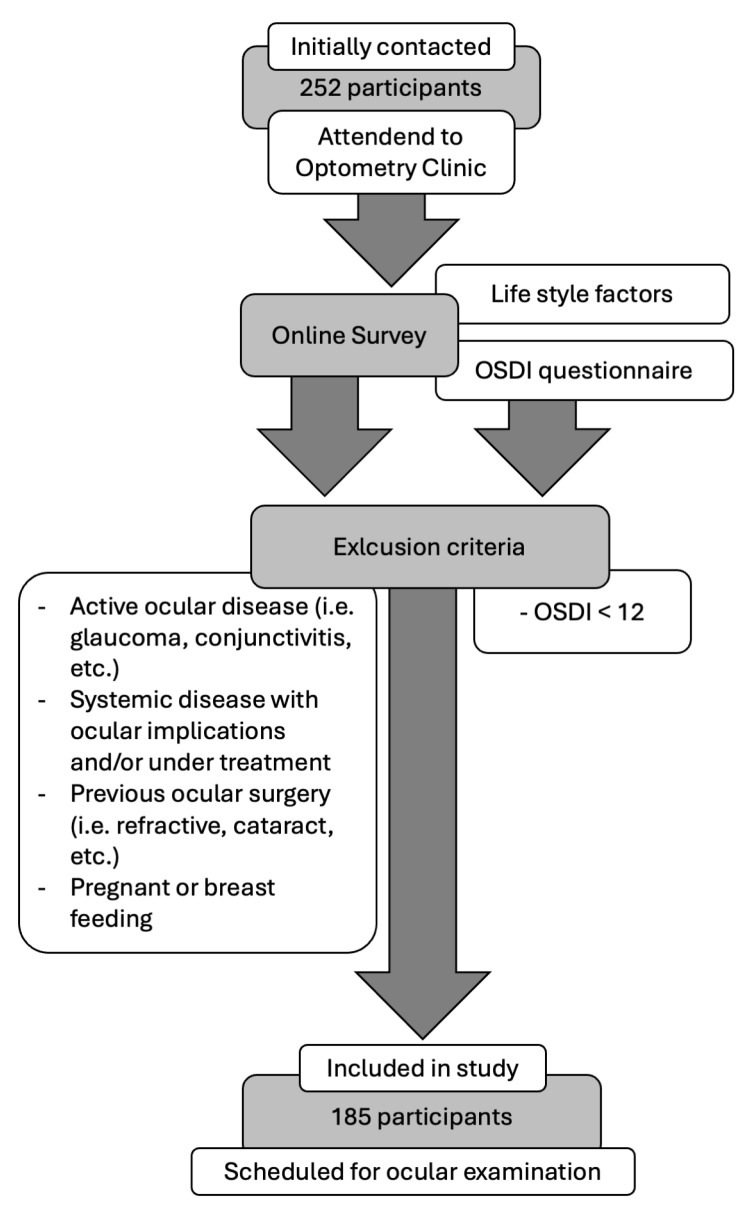
Inclusion and exclusion criteria flow chart.

The current cross-sectional study initially involved a total of 252 participants who attended the Optometry Clinic of the University of Santiago de Compostela for an eye test. Every participant completed a self-administered survey to collect information about ocular discomfort and lifestyle factors. This online survey was composed of an Ocular Surface Disease Index (OSDI) questionnaire and three extra questions regarding lifestyle factors (VDT use, CL wear, and eyedrop use) (Appendix A). Participants who showed OSDI values lower than 12 points were excluded from the study [19,20]. Also, participants with active ocular disease (i.e., glaucoma, conjunctivitis, etc.), systemic diseases with ocular implications (i.e., arthritis or thyroid disease), who were pregnant or breastfeeding, or who had undergone any eye surgery (i.e., glaucoma, cataract, refractive surgery, etc.) were excluded from the study [9]. Finally, 185 participants were scheduled to undergo a comprehensive ocular examination (Figure 1). This investigation adhered to the tenets of the Helsinki Principles and was approved by the institution’s bioethical committee under code number USC-40/2020. Every participant has signed a written informed consent form for their inclusion in the study.

### 2.2. Protocol and Procedures

Every participant was scheduled for a single appointment where a battery of ocular surface procedures was performed from the least to the most invasive test, and it was composed of tear film osmolarity, Fluorescein Break Up Time (FBUT), Maximum Blink Interval (MBI), and corneal staining. The protocol was executed in a controlled environment with regulated light, temperature (20–23 °C), and humidity (50–60%).

#### 2.2.1. Ocular Discomfort and Lifestyle Factors Online Survey

The online survey was elaborated on using Microsoft Forms software (Microsoft 365, Microsoft Corporation, Washington, DC, USA) and was composed of a full OSDI questionnaire and three extra questions regarding VDT use, CL wear, and eyedrop use. OSDI questionnaire is a validated form to assess ocular discomfort and its compatibility with dry eye symptomatology [21,22]. It is formed by 12 questions that assess the ocular symptomatology regarding different situations. VDT use was distributed into 4 categories depending on the number of hours using VDT that was self-precepted by the participant: (1) less than 4 h per day, (2) between 4 and 6 h per day, (3) between 6 and 8 h per day, and (4) more than 8 h per day. CL wear and eyedrop use were both recorded as “user” and “no user” (Appendix A).

#### 2.2.2. Tear Film Osmolarity

Tear film osmolarity was measured with the TearLab (Trukera Medical, Southlake, TX, USA) osmometer. This is an electric impedance osmometer with disposable cards that collects a small tear sample from each participant’s lower meniscus [23,24]. The instrument and test cards were stored in a temperature- and humidity-controlled room where the study was carried out. During all procedures, the same test card lot number was used.

#### 2.2.3. Tear Film Break-Up Time and Maximum Blink Interval

FBUT is defined as the first black spot, line, or area detected on a fluorescein-stained tear film when the participant is instructed to keep their eyes open without blinking. MBI is the maximum time that a participant can keep their eyes open, even after the tear film has already ruptured. To measure FBUT, fluorescein strips (Fluostrips, Contacare Opthalmics and Diagnostics, Vadodara, India) were placed in contact with the temporal conjunctiva, and a video was recorded using the multidiagnostic platform OCULUS Keratograph 5M (OCULUS GmbH, Wetzlar, Germany). This instrument features a module for capturing FBUT videos, projecting a beam of cobalt blue light over the participant’s ocular surface to enhance fluorescence visibility. Participants were properly positioned in front of the instrument and instructed to blink three times, followed by holding their eyes open until they could no longer do so. FBUT and MBI for each participant were recorded three times, and the videos were subsequently analyzed by a second masked observer using VLC open-source v. 3.0.20 software (VideoLAN Organization, Paris, France) [25]. Only the two closest measurements were used to calculate the mean values of FBUT and MBI, as recommended by previous reports [26].

#### 2.2.4. Corneal Staining

The corneal staining was immediately video recorded after measuring the FBUT, taking advantage of the fact that the ocular surface was already stained with fluorescein. This procedure was also video recorded with the fluorescein module of the OCULUS Keratograph 5M, and videos were analyzed by a second masked observer that quantified the ocular damage according to the Oxford grading scale [27,28]. The Oxford scheme classified the ocular staining into 5 severity grades: (0) No staining, (1) Mild, (2) Mild-Moderate, (3) Moderate, and (4) Severe [27].

### 2.3. Statistical Analysis

Statistical analysis was performed with the IBM SPSS v.29 software for MacOS (SPSS Inc., Chicago, IL, USA). Only the right eye of each participant was analyzed so as not to generate statistical overestimation [29]. In order to evaluate the implications of lifestyle factors on ocular surface parameters related to age, the sample was segregated into three population groups based on their age range. The age criteria were established as: Group 1—Less than 40 years old; Group 2—Between 40 and 60 years old; and Group 3—Older than 60 years old. The main justification for this group segregation was based on the progression of presbyopia and the associated lifestyle changes, such as dropout of CL or alterations in tear film [10,16,17,30,31].

First, the normality distribution of the continuous variables was checked by performing Kolmogorov-Smirnov test, which is recommended for samples bigger than 50 cases, and showed that OSDI, FBUT, MBI, and tear film osmolarity followed a non-normal distribution (Kolmogorov-Smirnov test, *p* ≤ 0.040). Due to the non-normal distribution of the data, it was transformed to logarithms for performing parametric one-way ANOVA and multiple linear regression. It was decided to apply a logarithmic transformation to the data to perform parametric tests, as these tests are statistically more robust. However, the data presented in the tables within the results section are in their original form to facilitate their clinical interpretation. Secondly, an ANOVA test between age groups for each variable was performed through the continuous variables in its logarithmic transformation, or crosstabs chi-square and Fisher’s exact tests were performed through categorical variables. Post hoc analyses between groups were performed following Bonferroni adjustment for one-way ANOVA. Thirdly, the influence of age and lifestyle habits (VDT use, CL wear, and eyedrop use) was checked by performing multiple linear regression. Finally, data was represented in boxplot graphs to visually understand the data managed.

## 3. Results

### 3.1. Descriptive Statistics and Differences Between Groups

**Figure 2:**
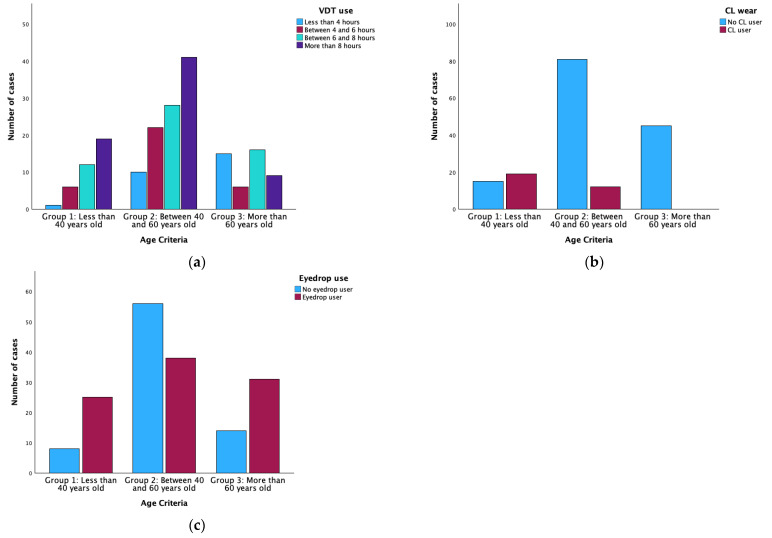
Distribution of lifestyle factors studied among the age criteria groups. (**a**) VDT is used according to age; (**b**) CL is worn according to age; (**c**) Eyedrop is used according to age. CL: Contact Lens; VDT: Video Display Terminal.

Table 1 represents descriptive statistics of the managed sample and differences between groups. When the sample was divided by age (Group 1 younger than 40 years old, Group 2 between 40 and 60 years old, and Group 3 older than 60 years old), there were found statistically significant differences between groups for OSDI, osmolarity, FBUT, MBI, VDTs use, CL wear, and eyedrop use (One-way ANOVA or Chi-square test, all *p* ≤ 0.029). However, there were no statistically significant differences in sex and corneal staining between groups (Chi-square test or Fisher’s exact test, both *p* ≥ 0.425). Paired analyses found differences between group 1 and group 2 in osmolarity, FBUT, CL wear, and eyedrop use (Bonferroni post hoc or Fisher’s exact test, all *p* ≤ 0.005); between group 1 and group 3 in OSDI, osmolarity, FBUT, MBI, VDT use, and CL wear (Bonferroni post hoc or Fisher’s exact test, all *p* ≤ 0.028); and between group 2 and 3 in VDT use, CL wear, and eyedrop use (Fisher’s exact test, all *p* ≤ 0.009). No other group showed statistically significant differences in the paired post hoc analysis (Bonferroni post hoc or Fisher’s exact test, all *p* ≥ 0.116). Figure 2 graphically represents how the lifestyle factors (VDT use, CL wear, and Eyedrop use) vary between age groups.

### 3.2. Correlations Between the Studied Parameters

Statistically significant correlations were found between corneal staining and osmolarity, FBUT, MBI, and eyedrop use (Spearman correlation; all r ≥ 0.149 and *p* ≤ 0.044; Table 2); between MBI and osmolarity, FBUT, and CL wear (Pearson or Spearman correlation; all r ≥ 0.150 and *p* ≤ 0.049; Table 2); and between osmolarity and FBUT, and CL wear (Pearson or Spearman correlation; both r ≥ 0.145 and *p* ≤ 0.050; Table 2).

### 3.3. Multiple Linear Regression of OSDI, Osmolarity, FBUT, and MBI Regarding the Age and Lifestyle Factors

Multiple linear regression was performed to investigate the relationship between OSDI, osmolarity, FBUT, or MBI with age criterion and lifestyle factors (VDT use, CL wear, and eyedrop use).

**Figure 3:**
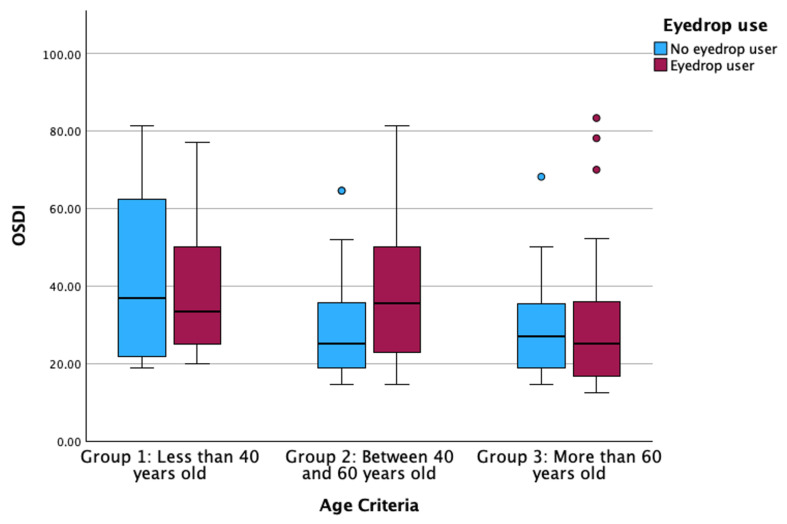
Boxplot of the OSDI distributed according to eyedrop use and the age criteria groups. OSDI: Ocular Surface Disease Index. The box illustrates the sample within the interquartile range (25th to 75th percentiles), while the black line indicates the median value. The dots signify outliers (values that fall more than 1.5 box lengths beyond the 25th or 75th percentiles).

For OSDI, the results showed a statistically significant relationship between eyedrop use and age criterion (Multiple linear regression; B = 0.065 for eyedrop use and B = −0.075 for age criterion, both *p* ≤ 0.040; Figure 3), while no other predictable variable showed statistically significant association (Multiple linear regression, all *p* > 0.347).

**Figure 4:**
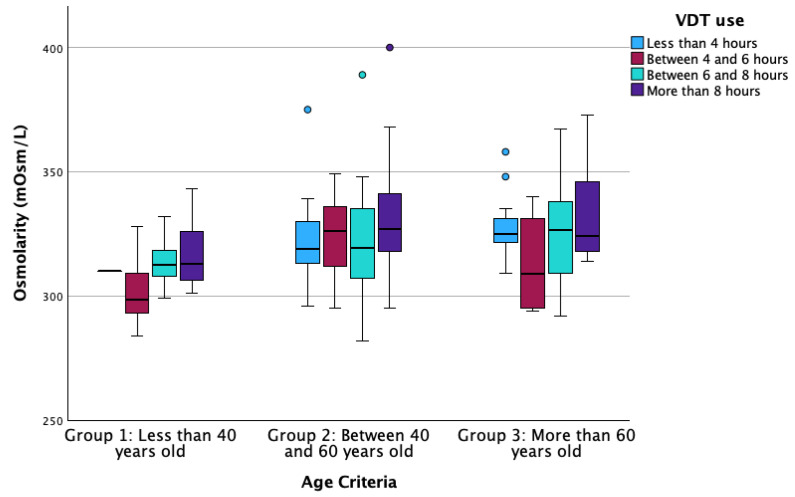
Boxplot of the osmolarity distributed according to VDT use grades and the age criteria groups. VDT: Video Display Terminal. The box illustrates the sample within the interquartile range (25th to 75th percentiles), while the black line indicates the median value. The dots signify outliers (values that fall more than 1.5 box lengths beyond the 25th or 75th percentiles).

In the case of osmolarity, the results showed that there exists a statistically significant relationship between VDT use and age criterion (Multiple linear regression; B = 0.004 for VDT use and B = 0.009 for age criterion, both *p* ≤ 0.038; Figure 4), while no other predictable variable showed statistically significant association (Multiple linear regression, all *p* > 0.484).

**Figure 5:**
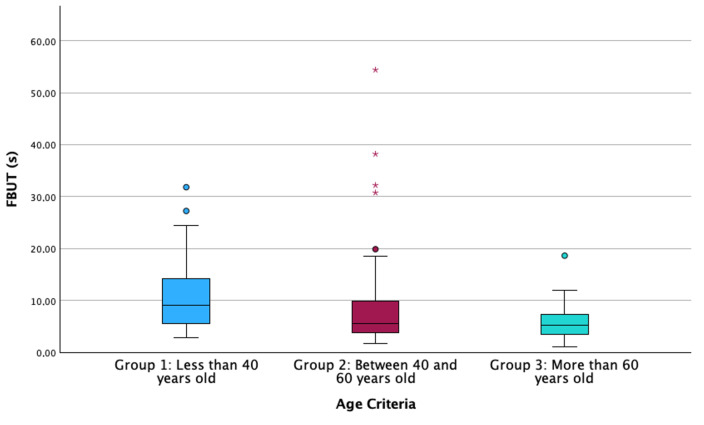
Boxplot of the FBUT distributed according to the age criteria into the 3 groups. FBUT: Fluorescein Break-Up Time. The box illustrates the sample within the interquartile range (25th to 75th percentiles), while the black line indicates the median value. The dots signify outliers (values that fall more than 1.5 box lengths beyond the 25th or 75th percentiles), and the asterisk denotes extreme outliers (values exceeding 3 box lengths from the 25th or 75th percentiles).

Results showed a statistically significant relationship between age criterion and FBUT (Multiple linear regression; B = −0.131, *p* = 0.001; Figure 5), while no other predictable variable showed a statistically significant association (Multiple linear regression, all *p* > 0.219).

**Figure 6:**
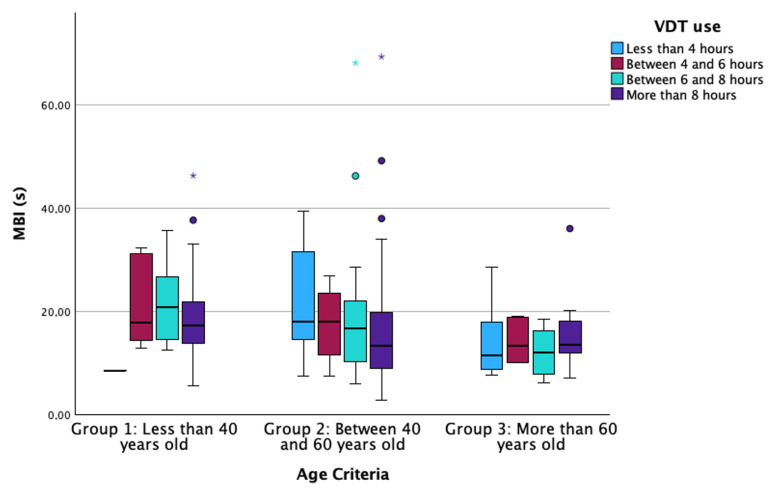
Boxplot of the MBI distributed according to VDT use grades and the age criteria groups. MBI: Maximum Blink Interval; VDT: Video Display Terminal. The box illustrates the sample within the interquartile range (25th to 75th percentiles), while the black line indicates the median value. The dots signify outliers (values that fall more than 1.5 box lengths beyond the 25th or 75th percentiles), and the asterisk denotes extreme outliers (values exceeding 3 box lengths from the 25th or 75th percentiles).

Regarding MBI, the results showed a statistically significant relationship between VDT use and age criterion (Multiple linear regression; B = −0.037 for VDT use and B = −0.303 for age criterion, both *p* ≤ 0.021; Figure 6), while no other predictable variable showed statistically significant association (Multiple linear regression, all *p* > 0.263).

## 4. Discussion

The aging process has been established as a factor that causes changes throughout the entire organism and impacts every individual in the world [32]. Overall, lifestyles have been changing for more than 50 years due to multiple factors, such as technological development, health and wellness trends, and social and cultural changes [33,34]. Healthier lifestyles have become more widespread in recent years, aiming for active aging and the enjoyment of a fulfilling life in later years [35,36]. However, some lifestyle factors could influence or alter these healthy habits, such as the high presence of screens in all jobs and their involvement in leisure time or the use of CLs, which can be beneficial for practicing some sports [34,37].

Excessive use of VDTs has been noticed to enhance ocular discomfort due to the alteration of different LFU structures [6,11,38,39,40,41,42]. Authors have identified a clear relationship between high exposure to VDTs and sleeping problems and obesity due to digital addiction [43,44]. Regarding ocular health, previous reports have identified diminished tear film stability values after long periods of screen use, independent of the measurement technique performed by the researchers, such as Non-Invasive Break-Up Time or FBUT [6,38,39,41,42]. The alteration of tear film stability results from a reduced blinking rate that occurs during activities requiring focus, such as screen use [6,11,42,45]. Additionally, the decrease in tear film stability is exacerbated by an increase in the frequency of incomplete blinks, which fail to adequately distribute the tear film across the ocular surface [40,46]. However, the multivariate regression analysis of the current manuscript showed that aging is the factor that significantly influences FBUT variation (Figure 5), rather than VDT, CL or eyedrop use. Additionally, FBUT was significantly different between groups, with younger participants showing higher FBUT values than older ones, similar to previous reports [47]. These results support the hypothesis that tear film stability decreases with aging. In addition, previous studies observed a decrease in tear metabolites (lysozyme, lipocalin and lactoferrin) associated with aging, although they failed to establish a direct relationship between this decrease and clinical parameters [48]. In terms of MBI, this ocular parameter quantifies the time between blinks in which the participant is not exposed to stimuli that would generate blinking, even though the tear film is not adequately covering the cornea. The current investigation found implications of age and VDT use on this parameter (Figure 6). This finding suggests that both age and VDT use influence stimuli perception, showing that in group 1 and group 2, those participants who use VDTs for more than 8 h per day have lower MBI values. However, participants in group 3 (more than 60 years old) showed lower MBI values compared to participants in the other groups (Figure 6), independent of the hours of VDT use. These results confirm that the use of VDTs disrupts ocular homeostasis and impairs corneal sensitivity due to prolonged exposure to elevated levels of tear metabolites. This, in turn, may lead to alterations in the sensitivity of the ocular surface due to aging and long exposure to homeostasis alteration [32,49]. Also, MBI has been found to be significantly lower in dry-eye participants compared to non-dry-eye controls [50], suggesting that altered ocular surface homeostasis affects ocular sensitization. Finally, the present investigation found a significant influence of age and VDT use on tear film osmolarity (Figure 4). Within each age group, participants who used VDTs for fewer hours showed lower osmolarity values than those who used VDTs for more hours. Additionally, older participants showed higher tear film osmolarity values than younger ones. This finding is supported by FBUT results, which indicate that participants with poor tear film stability and homeostasis alterations will show higher concentrations of inflammation mediators that increase tear film osmolarity. The measurement of tear film osmolarity has been identified as a reliable indicator of ocular homeostasis [20,32]. This investigation confirms that participants who use VDTs for extended periods exhibit altered ocular surface homeostasis, potentially due to a reduced blink rate and an increase in incomplete blinking during screen use.

Other factors like CL wear have both beneficial and harmful implications on the body. The use of CLs could beneficially influence the adoption of healthier lifestyles, such as participating in sports or social activities where wearing spectacles is uncomfortable. On the other hand, several authors have reported that wearing CLs for many years and not complying with the recommended usage guidelines (extending replacement times or not cleaning them properly) can lead to changes in the LFU [10,30,51,52]. However, the current investigation reveals no significant clinical implication of CL wear on any of the ocular parameters in the multiple linear regression when other factors were considered. Regarding lifestyle, statistically significant differences were found between age groups, showing that younger participants are more interested in wearing CLs than older ones (Figure 2b). Younger participants used to have a more active life than older ones, despite current trends promoting healthier lives among the elderly [17,53]. Wearing CLs could provide an optimal and comfortable vision in daily activities that require focusing on various distances. However, near vision is often compromised with the onset of presbyopia. Individuals with presbyopia who previously wore CLs frequently try multifocal CLs but report a reduction in vision quality at certain distances [17,30]. Furthermore, the cost of multifocal CLs is nearly double that of single-vision CLs. These factors, economic and visual quality, may account for the lower prevalence of CL wearers among older participants in this study [30].

The prevalence of eye discomfort is on the rise, attributable to a multitude of factors, including age, use of VDTs, wearing CL, maintenance of controlled humidity and temperature, and other factors [5,6,40,54,55]. Nevertheless, when all these factors are considered collectively, some may be identified as having greater significance than others. The current investigation found that ocular discomfort measured by the OSDI questionnaire could be influenced by age and the use of eyedrops (Figure 3). Eyedrops are widely prescribed to relieve ocular complaints due to CL wear or VDT use, as hydration of the ocular surface diminishes hyperosmolarity, which is the main cause of homeostasis loss [56]. The younger participants (Group 1) and older participants (Group 3) showed that no eyedrop users have higher OSDI values than eyedrop users. Conversely, eyedrop users in Group 2 showed higher symptoms than no eyedrop users. In this sense, participants who experience greater discomfort are also those who closely follow their eyecare practitioner’s recommendations to improve eye comfort, such as using eyedrops [57,58,59]. Overall, OSDI values of both users and no users of eyedrops vary according to age, with older participants showing lower OSDI values than younger participants. Finding that older participants have lower OSDI values indicates that neurotrophy and neuroadaptation occur due to continuous exposure to inflammatory triggers that alter the ocular surface [32,49,60].

Several limitations should be reported and discussed in the present investigation. First, only participants with positive values in the OSDI questionnaire due to ocular complaints were involved. Only these types of participants should be considered at the time of interpreting the current results; however, ocular discomfort is becoming increasingly prevalent in everyday clinical practice. Secondly, the sample was composed mainly of women, as occurs habitually in studies that involve ocular examinations. Women are more likely to develop eye diseases such as dry eye due to hormonal variations [13,61]. However, various strengths should be notably considered. On the one hand, only the right eye of each participant was included in the statistical analysis to avoid artificially enhancing the statistical power, even though the current manuscript evaluated both eyes of each participant. In contrast, multivariate regression provides information about which factors highly influence the alteration of each ocular parameter. This research provides an insightful view of the actual lifestyle regarding ocular and visual habits of the population, which may vary in 10 or 20 years. Habits and lifestyles always change, and time flows; people who are under 40 now will be in their 40s and 60s in a few years, along with their habits. For instance, individuals who wear CLs now may continue to wear them in the future, although some may choose alternative vision correction methods. The same could occur with the prolonged use of VDT or with the use of eyedrops. These authors encourage similar research to be conducted in the future to learn about the habits and lifestyles of the population.

## 5. Conclusions

In conclusion, although lifestyles are changing due to a more connected and health-conscious world, some factors that could alter the ocular surface have controversial implications. Age remains the most important factor influencing variations in symptomatology as well as in tear film osmolarity, FBUT and MBI. However, lifestyle factors such as VDT use or eyedrop usage may also influence ocular symptomatology, tear film osmolarity and MBI. Establishing a healthier relationship with digital device use, including scheduled breaks and ergonomic positioning, may help maintain ocular homeostasis and prevent ocular discomfort associated with these devices. When age is included in the equation, no implication of CL wear was found in any of the studied ocular parameters. Long-term CL use can lead to alterations in the ocular surface; however, this study did not identify contact lens users among older age groups.

## Figures and Tables

**Table 1:** Descriptive statistics of every studied parameter and differences between groups.

	N	Group 1 (<40 Years Old)	Group 2 (40–60 Years Old)	Group 3 (>60 Years Old)	*p*-Value
		38	101	46	
Sex (% women)	185	76.3%	83.2%	82.6%	0.666 ††
Age (Mean ± SD)	185	28.2 ± 1	51.7 ± 0.4	66.8 ± 0.7	**-**
OSDI values (Median [IQR])	185	33.3 [22.9–50]	27.5 [20.6–38.5]	25 [17.7–35.9]	0.029 *
Osmolarity (mOsm/L) (Median [IQR])	185	310 [303–318.5]	323 [314–337]	324 [314–338]	<0.001 *
FBUT (s) (Median [IQR])	185	9.4 [5.5–14.2]	4.9 [3.8–8.9]	5.1 [3.3–7.2]	<0.001 *
MBI (s) (Median [IQR])	185	20.3 [14.9–24.6]	15.44 [9–20]	12.6 [8.8–17.5]	0.006 *
Corneal staining (Oxford scheme) (Median [IQR])	185	0 [0–1]	1 [0–2]	1 [0–1.5]	0.425 †
VDT use (Median [IQR])	185	3 [3–4]	3 [2–4]	3 [1–3]	**<0.001 ††**
CL wear (Median [IQR])	172	1 [0–1]	0 [0–0]	0 [0–0]	**<0.001 ††**
Eyedrop use (Median [IQR])	172	1 [0.5–1]	0 [0–0]	1 [0–1]	**<0.001 ††**

* ANOVA test; † Fisher’s exact test; †† Chi-square test; CL: Contact Lens; Bold *p*-values are those statistically significant; FBUT: Fluorescein Break-Up Time; IQR: Interquartile Range; MBI: Maximum Blink Interval; OSDI: Ocular Surface Disease Index; SD: Standard Deviation; VDT: Video Display Terminal.

**Table 2:** Correlations between ocular parameters and factors.

		Osmolarity	FBUT	MBI	Corneal Staining	VDT Use	CL Wear	Eyedrop Use
OSDI	r	−0.018 *	−0.005 *	−0.006 *	0.017 †	0.047 †	0.086 †	0.143 †
	*p*	0.811	0.947	0.932	0.814	0.523	0.262	0.061
Osmolarity	r		−0.145 *	−0.150 *	**0.149 †**	0.072 †	**−0.155 †**	0.008 †
	*p*		**0.050**	**0.042**	**0.042**	0.328	**0.043**	0.921
FBUT	r			0.719 *	**−0.268 †**	0.011 †	0.139 †	0.077 †
	*p*			**<0.001**	**0.001**	0.881	0.069	0.313
MBI	r				**−0.154 †**	−0.037 †	**0.150 †**	0.066 †
	*p*				**0.036**	0.618	**0.049**	0.386
Corneal Staining	r					−0.056 †	−0.047 †	**−0.154 †**
	*p*					0.450	0.537	**0.044**
VDT use	r						0.123 †	−0.028 †
	*p*						0.107	0.716
CL wear	r							0.090 †
	*p*							0.240

* Pearson test; † Spearman test; Bold *p*-values are those statistically significant; OSDI: Ocular Surface Disease Index; CL: Contact Lens; FBUT: Fluorescein Break-Up Time; MBI: Maximum Blink Interval; VDT: Video Display Terminal.

**Figure A1:**
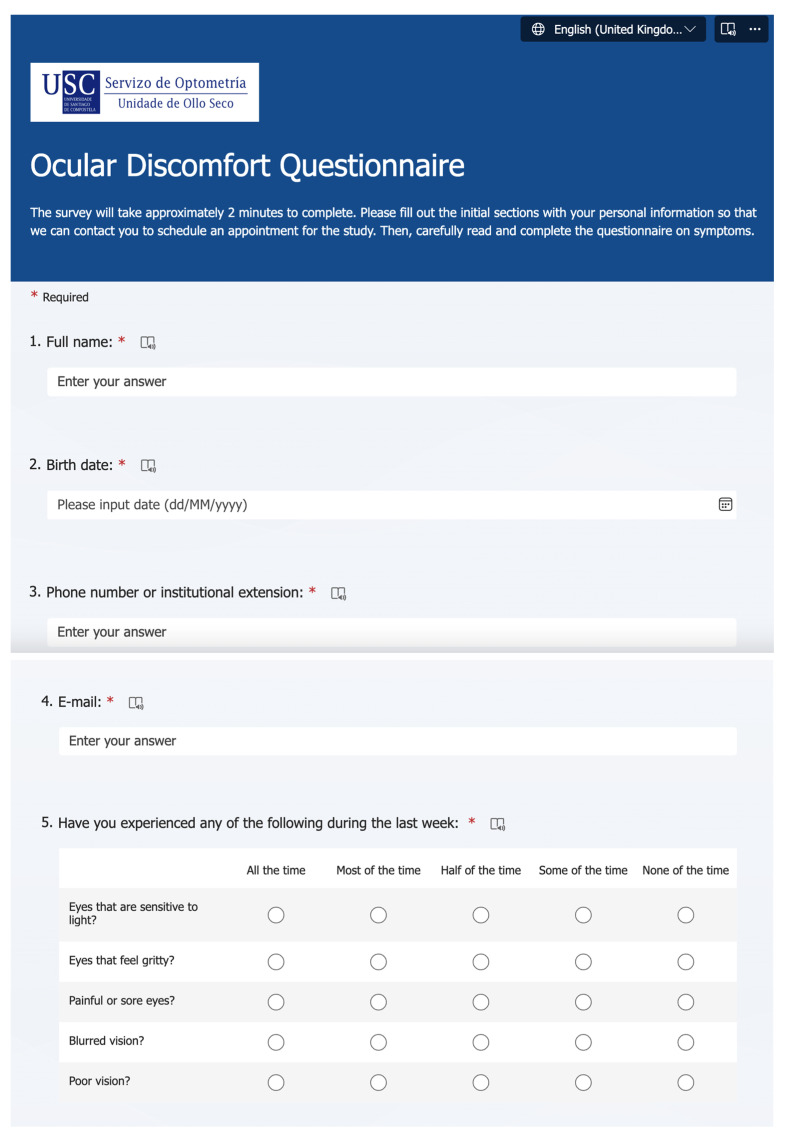

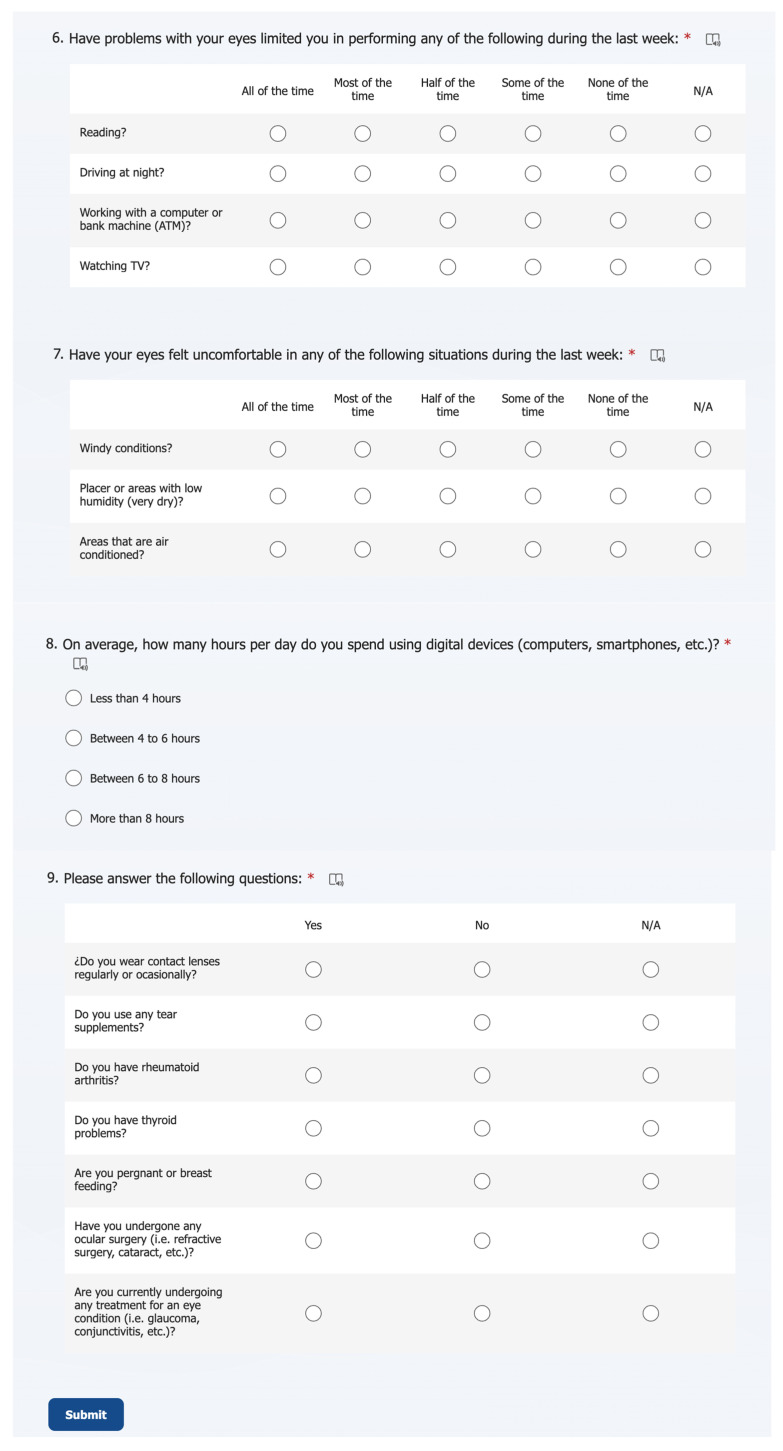
Online self-administered survey to collect information about ocular discomfort and lifestyle factors. Access to the questionnaire following the current link: https://forms.office.com/e/3rK1HrEDXz.

## Data Availability

Data is unavailable due to privacy restrictions.
